# Probing Nanomechanics by Direct Indentation Using Nanoendoscopy-AFM Reveals the Nuclear Elasticity Transition in Cancer Cells

**DOI:** 10.1021/acsanm.5c03044

**Published:** 2025-10-12

**Authors:** Takehiko Ichikawa, Yohei Kono, Makiko Kudo, Takeshi Shimi, Naoyuki Miyashita, Tomohiro Maesaka, Kojiro Ishibashi, Kundan Sivashanmugan, Takeshi Yoshida, Keisuke Miyazawa, Rikinari Hanayama, Eishu Hirata, Kazuki Miyata, Hiroshi Kimura, Takeshi Fukuma

**Affiliations:** † Nano Life Science Institute (WPI-NanoLSI), 12858 Kanazawa University, Kakuma-machi, Kanazawa, Ishikawa 920-1192, Japan; ‡ Division of Electrical Engineering and Computer Science, Kanazawa University, Kakuma-machi, Kanazawa, Ishikawa 920-1192, Japan; § Division of Tumor Cell Biology and Bioimaging, Cancer Research Institute of Kanazawa University, Kakuma-machi, Kanazawa, Ishikawa 920-1192, Japan; ∥ Biochemistry and Molecular Biology, University of Maryland School of Medicine, 655 W. Baltimore Street, Baltimore, Maryland 21201, United States; ⊥ Department of Immunology, Kanazawa University Graduate School of Medical Sciences, Kakuma-machi, Kanazawa, Ishikawa 920-1192, Japan; # Cell Biology Center, Institute of Integrated Research, Institute of Science Tokyo, 4259 Nagatsuta-cho, Midori-ku, Yokohama 226-8501, Japan; ¶ Graduate School of Bioscience and Biotechnology, Institute of Science Tokyo, 4259 Nagatsuta-cho, Midori-ku, Yokohama 226-8501, Japan

**Keywords:** atomic force microscopy, nanoendoscopy-AFM, nuclear elasticity, histone modification, chromatin compaction, epithelial-mesenchymal transition, serum

## Abstract

The assessment of nuclear structural changes is considered a potential biomarker of metastatic cancer. However, accurately measuring nuclear elasticity remains challenging. Traditionally, nuclear elasticity has been measured by indenting the cell membrane with a bead-attached atomic force microscopy (AFM) probe or aspirating isolated nuclei with a micropipette tip. However, indentation using a bead-attached probe is influenced by the cell membrane and cytoskeleton, while measurements of isolated nuclei do not reflect their intact state. In this study, we employed Nanoendoscopy-AFM, a technique in which a nanoneedle probe is inserted into a living cell to directly measure nuclear elasticity and map its distribution. Our findings show that nuclear elasticity increases under serum depletion but decreases when serum-depleted cells are treated with TGF-β, which induces epithelial–mesenchymal transition (EMT). Furthermore, we found that changes in nuclear elasticity correlate positively with trimethylation levels of histone H4 at lysine 20, rather than with nuclear lamins expression levels. These findings suggest that alterations in chromatin structure underlie changes in nuclear elasticity during the progression of cancer.

## Graphical abstract

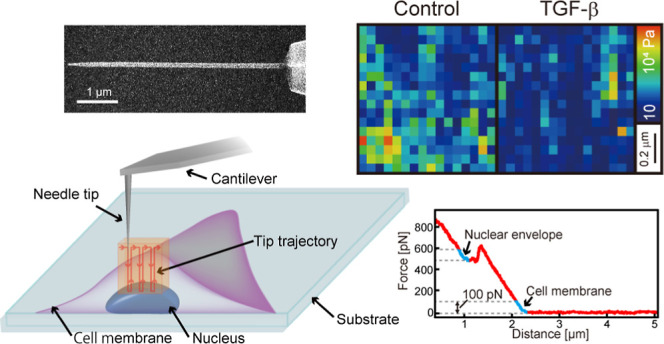

## Introduction

Nuclear mechanics influence gene regulation, division, and motility.^1–3^ Consequently, altered nuclear mechanics, such as nuclear elasticity, are a hallmark of diseases, including cancer progression.^4–8^ Recent studies have attempted to utilize changes in nuclear elasticity for cancer diagnosis. However, this remains challenging because traditional methods lack sufficient accuracy for assessing intact nuclear elasticity. For example, two major methods have been employed to measure nuclear elasticity: indenting the cell membrane above a nucleus using a colloidal probe cantilever in atomic force microscopy (AFM) or measuring isolated nuclei using AFM or microaspiration.^3,9–22^ The former method assesses not only nuclear elasticity but also contributions from the cell membrane and cytoskeleton. Meanwhile, the latter method does not necessarily reflect the properties of an intact nucleus. Additionally, methods utilizing magnetic beads or optical tweezers cannot apply large forces and may fail to measure elasticity accurately.^23,24^ Therefore, a new, more precise method is needed to accurately measure the elasticity of intact nuclei.

Recent advances in AFM technology have demonstrated that AFM with a nanoneedle probe can measure the mechanical properties of the nucleus in living cells. Nanoneedle-based AFM technology was first developed by Nakamura and colleagues and has been applied to molecular delivery and detection inside living cells.^25,26^ Subsequently, Sun’s group showed that nuclear elasticity could be measured inside a living cell using a nanoneedle probe.^27,28^ In their study, nuclear elasticity was estimated from force curves obtained only a few times without precise control over the probe’s position relative to the cell nucleus. A major limitation of this approach is the difficulty in avoiding obstacles, such as the cytoskeleton, vesicles, and other structures between the nuclear and the cell membranes, which compromises the accuracy of nuclear elasticity measurements. This is a critical issue because the primary advantage of nanoneedle-based AFM technology is its ability to measure intact nuclear elasticity while minimizing the influence of non-nuclear components.

The first objective of this study was to overcome these limitations by establishing a robust and reliable method for applying nanoneedle-based AFM to assess nuclear elasticity. We have developed a method named “Nanoendoscopy-AFM (NE-AFM)”, which allows for thousands of probe insertions into a cell without causing severe damage, achieved by optimizing the tip shape, refining insertion conditions, and developing dedicated analysis software.^29–31^ This technique enables nanoscale mapping of nuclear surface elasticity. It also improves the accuracy of nuclear elasticity measurements by selecting force curves that specifically reflect interactions with the nuclear membrane from a large data set.

With this validated NE-AFM platform, our second objective was to investigate how nuclear elasticity is dynamically regulated during key events in cancer progression. A pivotal process in cancer metastasis is the epithelial–mesenchymal transition (EMT), where cells acquire migratory, invasive properties. However, the impact of EMT on the mechanical properties of the intact nucleus has not been investigated. Therefore, we applied NE-AFM to quantify, for the first time, the changes in nuclear elasticity in living cells undergoing TGF-β-induced EMT, as well as in response to serum depletion.

Nuclear elasticity is thought to be influenced by two main factors: the nuclear envelope and chromatin compaction.^11,32,33^ The nuclear envelope consists of double phospholipid bilayers—the inner and outer nuclear membranes (INM and ONM)—and associated proteins.^34^ A part of heterochromatin is anchored to the nuclear lamina lining the INM. The nuclear lamina is connected to the cytoskeletal system through the linker of the nucleoskeleton and cytoskeleton (LINC) complex, which is localized to the INM and ONM and the lumen between them.^35^ The major structural components of the nuclear lamina are nuclear lamins, classified into A-type lamins (lamins A and C) and B-type lamins (lamins B1 and B2).^36,37^ These lamins assemble into filaments to form the nuclear lamina meshwork.^38–41^

Chromatin can be divided into heterochromatin (tightly packed) and euchromatin (loosely packed).^42^ Heterochromatin is characterized by specific modifications, such as histone H3 lysine 9 dimethylation and trimethylation (H3K9me2 and H3K9me3, respectively), lysine 27 trimethylation (H3K27me3), and H4 lysine 20 trimethylation (H4K20me3), which mark transcriptionally inactive regions.^43–46^ A decreased level of H3K9me3 and depletion of heterochromatin protein 1 (HP1) α cause nuclear softening.^33^

In this study, we first establish the utility of NE-AFM for high-accuracy analysis of nuclear elasticity in living cells. We then use this method to investigate changes in nuclear elasticity in response to serum status and during EMT. Finally, we explore whether these mechanical changes correlate with alterations in nuclear lamina and chromatin states.

## Results

### Whole-Cell Measurement Using NE-AFM

Figure 1a illustrates the process of whole-cell measurement using 3D NE-AFM. This technique repeatedly inserts a nanoneedle probe into the cell to measure the force versus distance (*F*-*z*) at arrayed-*xy* positions across the target area. The nanoneedle probe was fabricated by electron beam deposition (EBD) on the truncated tip of a commercial cantilever (BL-AC40TS-C2, spring constant: 0.1 N/m), as shown in Figure 1b. The nanoneedle length of 5 μm is long enough to measure the elasticity of the nuclear surface inside the living cell. The diameter of the needle was kept below 200 nm, as previous studies confirmed that nanoneedles with diameters smaller than this do not cause cell death following NE-AFM observation.^29,47^ For this experiment, the nanoneedle diameter was approximately 160 nm, well below this viability threshold (Figure 1b). The tip radius was approximately 24 nm.

**Figure 1:**
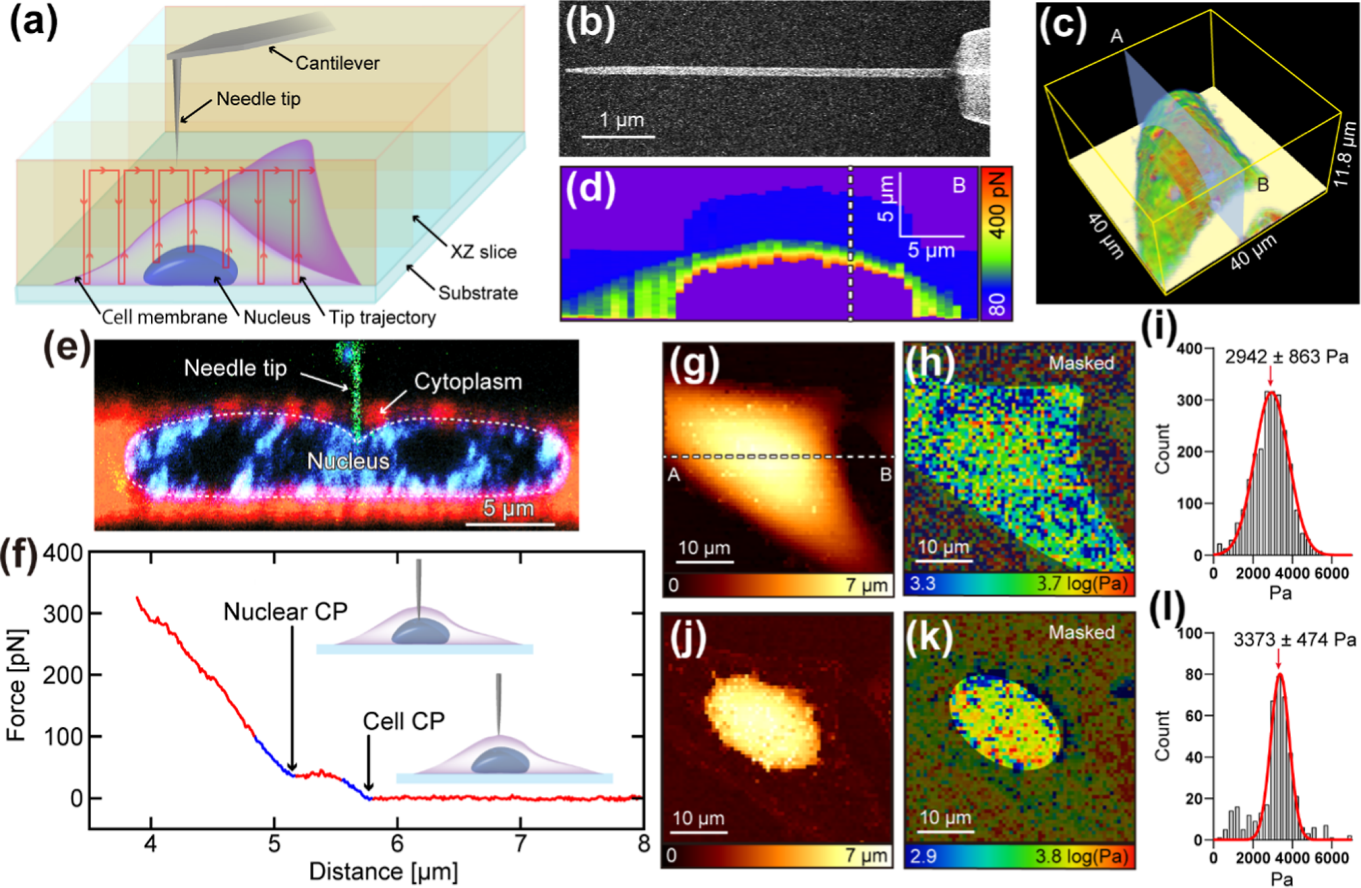
Nanoendoscopy-AFM (NE-AFM) measurement of a whole cell. (a) Schematic illustration of the NE-AFM applied to a whole cell. (b) Scanning electron microscope image of nanoneedle probe. (c) Volume rendering of the obtained 3D force map with a force-weighted transparency filter to visualize cell membrane surfaces. (d) A cross-sectional force map was taken along line AB in panel (c,g). (e) Cross-sectional image showing the tip pressing against the nucleus. The nanoneedle probe, cytoplasm, and nucleus were stained green, red, and blue, respectively. (f) Typical force–distance (*F*-*z*) curve during tip approach. The force represents the force applied to the tip, while the distance indicates the relative tip height with respect to the arbitrarily determined zero position. Cell contact point (Cell CP) and nuclear contact point (Nuclear CP) are marked. (g) Cell CP map. (h) Young’s modulus (*E*_Y_) map of the cell membrane. (i) Histogram of *E*_Y_ values for the cell membrane (mean value of the Gaussian fit: 2942 ± 863 Pa). (j) Nuclear CP map. (k) *E*_Y_ map of the nuclear surface. (l) Histogram of *E*_Y_ values for the nuclear surface (mean value of the Gaussian fit: 3373 ± 474 Pa).

Figure 1c presents a volume-rendered cell surface image constructed from the obtained 3D force map with a force-weighted transparency filter. Figure 1d depicts the cross-sectional force map along the line A-B in Figure 1c,g. Figure 1e displays the cross-sectional fluorescence image of the deformed nucleus during its indentation by the needle probe. A typical *F*-*z* curve is displayed in Figure 1f, where “distance” refers to the relative tip height from an arbitrarily determined zero position, and “force” is the vertical force applied to the tip. The *F*-*z* curve reveals several key events. As the tip approaches the cell, it first makes contact with and indents the cell membrane. This event, denoted as the cell contact point (Cell CP), is identified by a local rise in the force curve at approximately 5.8 μm (highlighted by the right blue line). Subsequently, the tip penetrates the membrane, which is indicated by a plateau in the force between 5.0 and 5.5 μm. Finally, the tip contacts and indents the nucleus at approximately 5.0 μm (the nuclear contact point, Nuclear CP). By fitting a modified Hertz model (for a paraboloidal indenter) to the corresponding indentation profile in the force curve, the Young’s modulus (*E*_Y_) of the cell membrane and the nucleus was estimated separately.^48,49^ To confirm that these measurements were not influenced by the underlying substrate, the bottom effect was also investigated and found to be minimal for both cell membrane and nuclear elasticity (Supporting Information, Figure S1).

By extracting data from the 3D force map, we can reconstruct the cell membrane’s CP and elasticity maps (Figure 1g,h). Figure 1i presents a histogram of the cell membrane’s *E*_Y_ (peak value: 2942 ± 863 Pa (*R* ^2^: 0.9873). Similarly, we can reconstruct nuclear CP and elasticity maps (Figure 1j,k), with Figure 1l showing the *E*_Y_ histogram for the nucleus (peak value: 3373 ± 474 Pa (*R* ^2^: 0.9411). Analyses were performed using custom software developed in-house.^31^

### Measurement of the Nuclear Elasticity of Cancer Cells with and without Serum

Previous studies have reported that chromatin states modulate nuclear elasticity and that serum influences these chromatin states.^50–52^ To investigate the effects of serum depletion on nuclear elasticity, we measured the elasticity of the intact nuclear surface in human lung cancer cells (PC9, harboring the EGFR Δexon19).^53^ Figure 2a illustrates the NE-AFM method used for these measurements, where 256 force curves were taken at 16 × 16 arrayed-*xy* positions over a 1 × 1 μm^2^ area around the nucleus center. The set point of the force curve measurements (0.6–0.8 nN) was determined such that the tip approach stops at the nuclear surface, minimizing the risk of tip or cell damage. Thus, a two-dimensional map of the lowest tip heights corresponds to a height map of the nuclear surface, as shown in Figure 2b.

**Figure 2:**
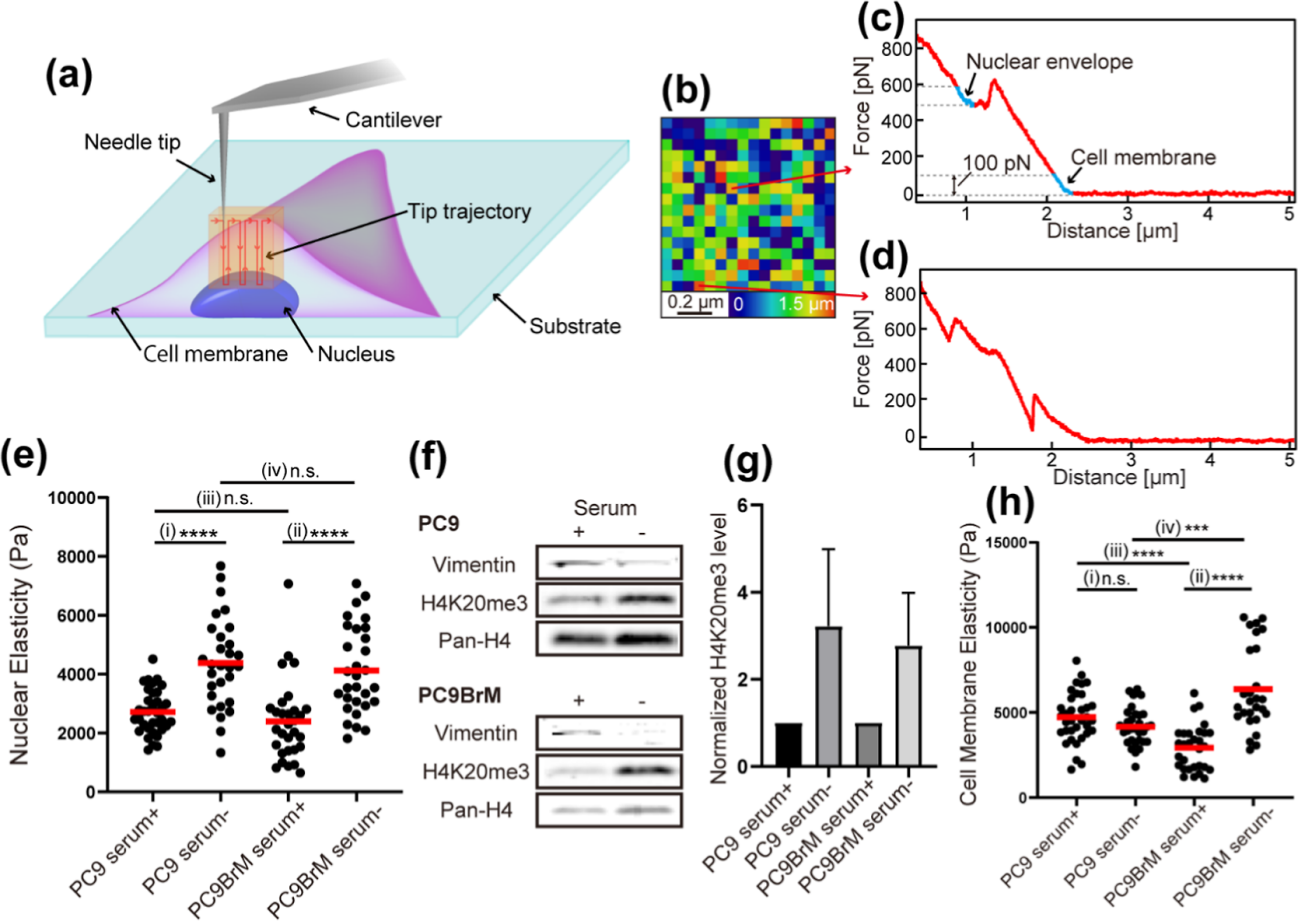
Quantitative analysis of nuclear elasticity by NE-AFM. (a) Schematic illustration of NE-AFM measurement on a nuclear surface. (b) Height map of the nucleus. (c) An example of a “good” force curve allowing unambiguous determination of the cell and nuclear CPs. (d) An example of a “bad” force curve is where identification of the cell or nuclear CP is difficult. (e) The distribution of nuclear elasticity of PC9 and PC9-BrM cells in normal (serum+) and serum-depletion (serum−) conditions. (f) Representative expression levels of vimentin, H4K20me3, and pan-H4 in PC9 and PC9-BrM with and without serum. (g) Bar graph of quantified levels of H4K20me3 normalized to pan-H4 from (f) by densitometry. Each bar was normalized to the expression level under serum + conditions in PC9 and PC9-BrM, respectively. (h) Distribution of cell membrane elasticity in PC9 and PC9-BrM cells under serum+ and serum-conditions.

Among the obtained 256 curves, some displayed a clear peak corresponding to cell membrane penetration, followed by a plateau and a sharp increase indicative of nuclear indentation (Figure 2c). For such “good” curves, we could reliably identify the cell and nuclear CPs and estimate *E*_Y_. However, some “bad” curves show multiple small peaks due to the interaction with other intracellular components, making it difficult to reliably identify CPs and estimate *E*_Y_ (Figure 2d). Therefore, we manually selected 20 good curves from the 256 curves and estimated the cell and nucleus *E*_Y_ by fitting the modified Hertz model for paraboloidal indenter to the indentation profiles (blue lines in Figure 2c) using a fixed force range of 100 pN. The indentation depth of the nucleus ranged from 150 to 250 nm. As this range is comparable to the thickness of the nuclear envelope and associated chromatin structures,^54–56^ the measurements should largely reflect their elasticity.

We measured the nuclear elasticity of living PC9 cells in culture media with and without serum. After 2 days of serum-free culture, nuclear elasticity significantly increased compared to conditions with serum (Figure 2e­(i) and Supporting Table S1, *p* < 0.0001; with serum: 2714 ± 126 Pa (average ±standard error of the mean, *N* = 35); without serum: 4384 ± 284 Pa (*N* = 29)). We then investigated potential factors contributing to this increase in nuclear elasticity. First, we examined the role of the cytoskeleton, but found that the expression level of β-actin did not change between cells cultured with and without serum (Supporting Information, Figure S2). Next, we hypothesized that this increase in nuclear elasticity was due to changes in chromatin compaction. To test this hypothesis, we measured the levels of H4K20me3, a marker that increases with chromatin compaction, via immunoblotting.^22,51^ Consistent with our hypothesis, H4K20me3 levels significantly increased under serum-depleted conditions compared to those before the serum depletion (Figure 2f,g, 3.22 ± 1.76 times higher than the serum-containing condition (*N* = 3)), indicating that serum depletion enhances chromatin compaction, leading to increased nuclear elasticity.

We performed similar experiments on brain-metastatic cells (PC9-BrM), which were established after four cycles of intracardiac injection and cancer cell collection.^57^ Through these brain metastasis cycles, PC9 lung cancer cells likely acquired the ability to invade the blood–brain barrier (BBB), a network of endothelial cells with continuous tight junctions that represents the rate-limiting step in the development of brain metastasis.^58,59^ To go through the tight channels, these brain-metastatic cells may acquire reduced nuclear elasticity, as previously reported for other cancer cells.^60^ Consistent with PC9 cells, serum depletion increased nuclear elasticity in PC9-BrM cells (Figure 2e­(ii), *p* < 0.0001; with serum: 2394 ± 249 (*N* = 30); without serum: 4122 ± 269 (*N* = 30)). H4K20me3 levels also increased under serum-depleted conditions (Figure 2f,g, 277 ± 1.21 (*N* = 3)). Unexpectedly, no significant differences in nuclear elasticity were observed between PC9 and PC9-BrM cells, regardless of serum presence (Figure 2e­(iii), *p* = 0.47; Figure 2e­(iv), *p* = 0.06).

Figure 2h presents the results of cell membrane elasticity. In PC9 cells, cell membrane elasticity did not differ significantly between the presence and absence of serum (Figure 2h­(i), *p* = 0.08; with serum: 4722 ± 250 (*N* = 35); without serum: 4166 ± 198 (*N* = 31)). However, in PC9-BrM cells, cell membrane elasticity was significantly higher under serum-depleted conditions than under normal serum-treated conditions (Figure 2h­(ii), *p* < 0.001; with serum: 2929 ± 241 (*N* = 30); without serum: 6371 ± 477 (*N* = 27)). Vimentin is one of the intermediate filament proteins distributed in the cytoplasm and is known to act as an elastic material of the cell. Increased expression levels of vimentin have been reported to enhance cell elasticity.^61,62^ However, under serum-depleted conditions in our experiment, vimentin expression was reduced (Figure 2f), indicating that vimentin is not the main factor in increased cell membrane elasticity in PC9-BrM cells. When comparing PC9 and PC9-BrM cells, PC9-BrM cells exhibited lower cell membrane elasticity than PC9 cells in the presence of serum but higher elasticity under serum-free conditions (Figure 2h­(iii), *p* < 0.0001 (with serum); Figure 2h­(iv), *p* < 0.001 (without serum)). This means that PC9-BrM cells respond differently to serum compared with PC9, which may partially explain the discrepancy regarding previous cell elasticity experiments.^63–67^

### EMT Induction Using TGF-β and Expression of Lamins and Histone Modifications

Malignant transformation of cancer often requires epithelial–mesenchymal transition (EMT) induction.^44–46,68^ To explore the relationship between malignant transformation and nuclear elasticity transitions, we examined whether EMT induction alters nuclear elasticity. EMT was induced by adding transforming growth factor (TGF)-β to a serum-free medium.^45,46^ EMT induction was confirmed by the upregulation of vimentin and N-cadherin (Figure 3a). Nuclear elasticity significantly decreased following EMT induction compared to control (serum-free) cells (Figure 3b, *p* = 0.0018; 3201 ± 197 Pa (*N* = 30)). A representative elasticity map of the nuclear surface in control and TGF-β-treated cells on a 1 × 1 μm^2^ area is shown in Figure 3c. In contrast, cell membrane elasticity remained unchanged (Figure 3d, *p* = 0.513; 4097 ± 302 Pa (*N* = 30); the elasticity map is shown in Figure 3e).

**Figure 3:**
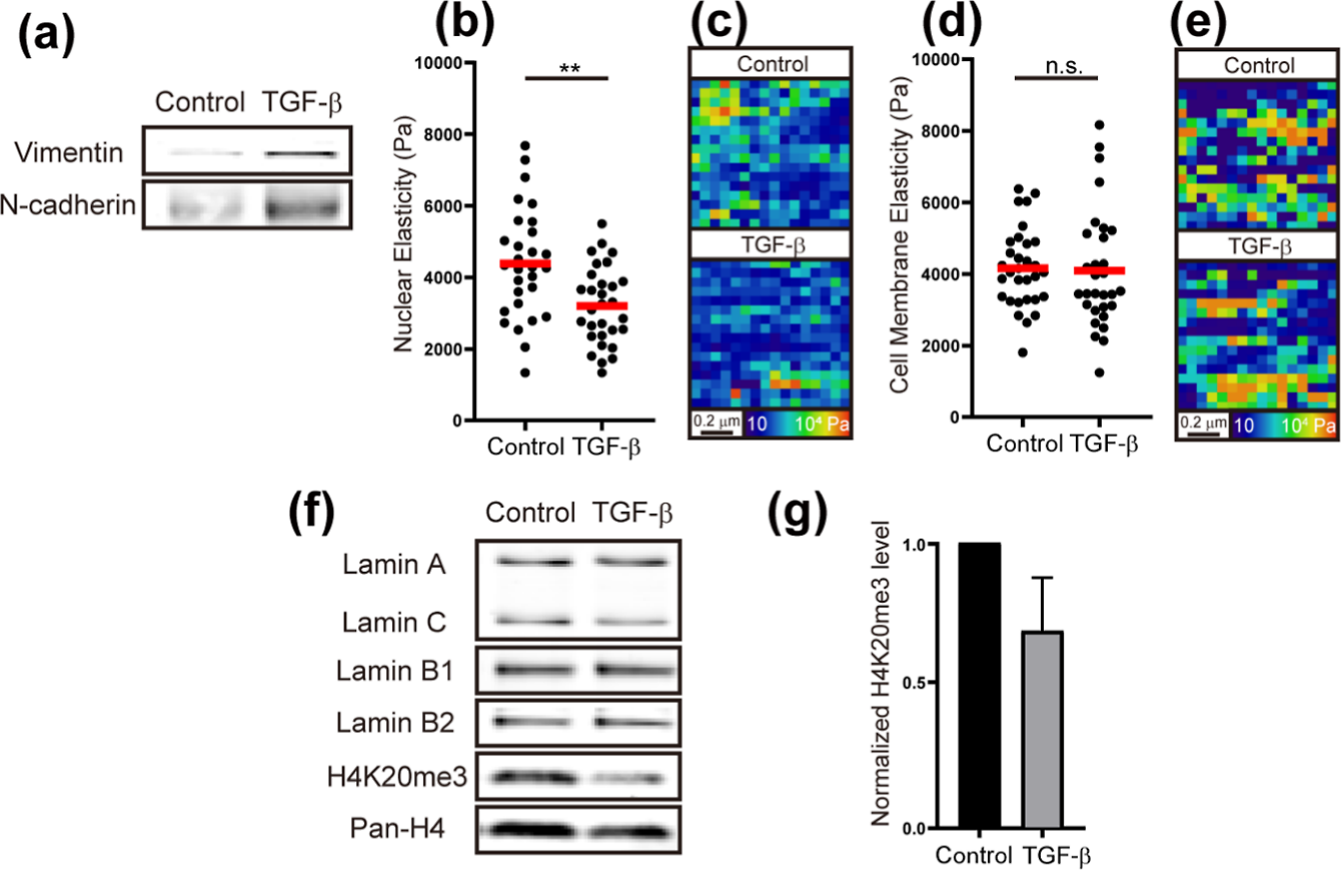
Measurement of the effect of EMT induction by TGF-β on nuclear and cell elasticity, as well as on lamins and histone modifications. (a) Expression levels of EMT markers (vimentin and N-cadherin), (b) Nuclear elasticity of control (serum-free) and TGF-β-treated cells. (c) Representative elasticity map of control and TGF-β-treated cell nuclear surface. (d) Cell membrane elasticity. (e) Representative elasticity map of the cell membrane. (f) Expression levels of lamins, H4K20me3, and pan-H4. (g) Densitometrically quantified graph of H4K20me3 normalized to pan-H4 from (f).

Previous studies have shown that the nuclear lamina plays a crucial role in regulating nuclear elasticity.^11,50,69–71^ To determine whether the expression levels of lamins (lamin A/C, B1, and B2) are affected by TGF-β treatment, we measured their expression levels by immunoblotting. As shown in Figure 3f, the expression levels of lamin A/C, B1, and B2 remained unchanged between control and TGF-β-treated cells. This suggests that lamins are not the primary factor regulating nuclear elasticity under these conditions. In contrast, H4K20me3 levels decreased in PC9 cells after EMT induction (Figure 3f,g, 0.69 ± 0.18 (*N* = 3)). These results support the hypothesis that chromatin compaction contributes to alterations in nuclear elasticity.

### Measurement of Nuclear Volume, Nuclear Deformation, and Cell Adhesion Area

We investigated the effects of serum and TGF-β on nuclear volume, nuclear deformation, and cell spreading to assess morphological changes in the cells. Figure 4a shows fluorescence images of nuclei under serum treatment, serum depletion, and TGF-β treatment, while Figure 4b,c present the distribution of nuclear volume and circularity under each condition. The nuclear volume of serum-depleted PC9 cells was significantly smaller than that of serum-treated cells. This reduction in nuclear volume may lead to increased nuclear elasticity by raising the concentration of structural components in the nuclear envelope. In contrast, no significant differences in nuclear volume were observed between serum-depleted cells and TGF-β-treated cells. Circularity, calculated by 4π × (area/perimeter^2^), quantifies how closely the shape resembles a perfect circle and serves as an indicator of nuclear deformation.^72^ The results showed no significant differences in circularity under these three conditions. Figure 4d presents representative fluorescence images of the cells under each condition, while Figure 4e shows the distribution of cell adhesion area (the 2D projected area of a cell). The cell adhesion area was significantly reduced after serum depletion and increased following TGF-β-treatment, likely due to enhanced lamellipodia spreading in response to serum and TGF-β. Overall, the presence or absence of serum and TGF-β did not significantly affect the shape of the nuclei or cells, nor did these factors influence the stiffness of the nuclear membrane.

**Figure 4:**
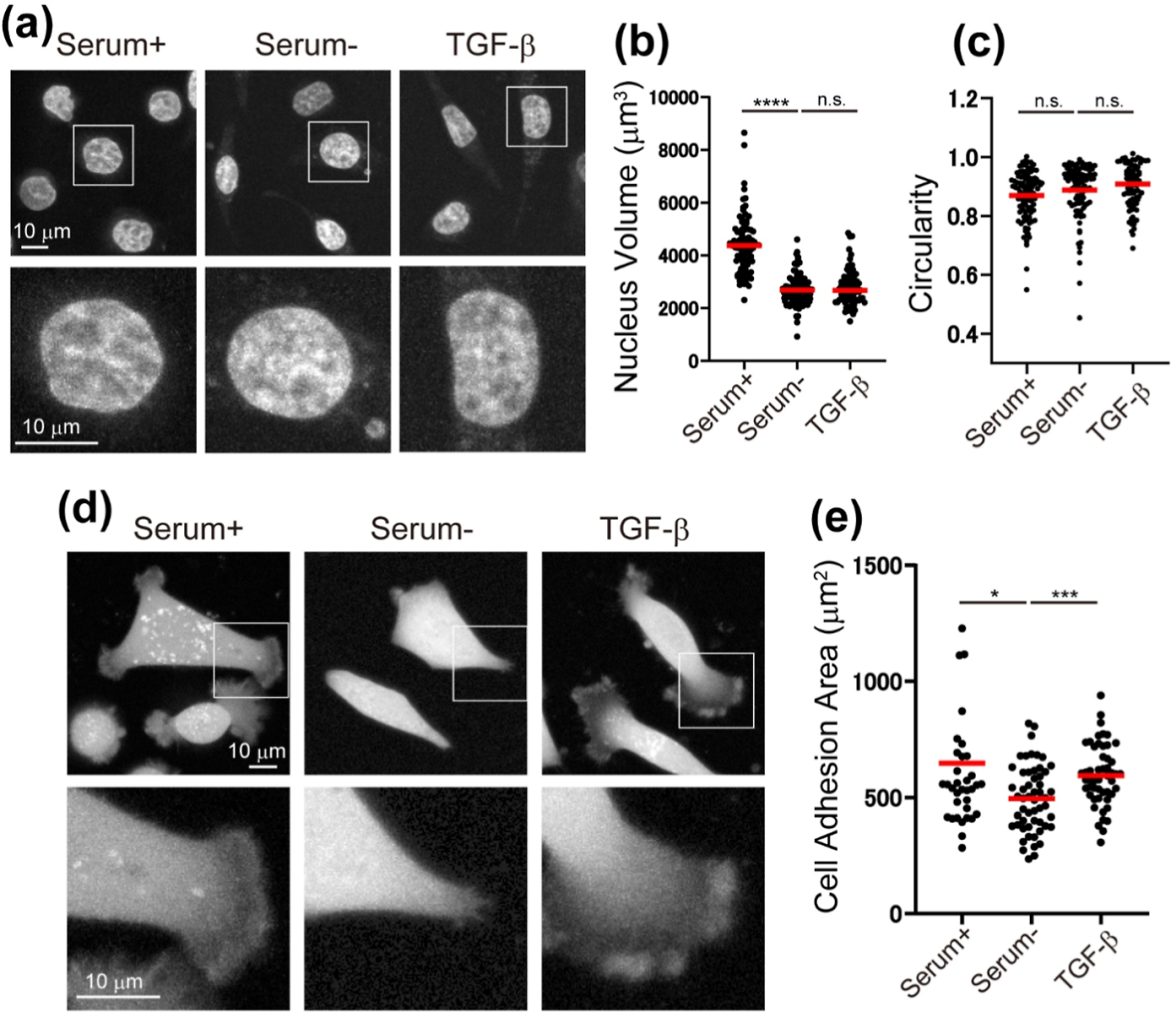
Quantification of nuclear volume, nuclear deformation, and cell adhesion area under serum (Serum+), serum depletion (Serum−), and TGF-β treatment. (a) Representative fluorescence images of nuclei for each treatment condition are shown as maximum-intensity projections. The images in the bottom row are magnified views of the area indicated by white boxes. (b) Distribution of nuclear volume. (c) Distribution of the nuclear circularity. (d) Representative fluorescence images of the cell under serum-containing medium, serum depletion, and TGF-β treatment. (e) Distribution of cell adhesion area.

## Discussion

In this study, we employed NE-AFM, which we recently developed to measure the nanoscale mechanical properties of nuclear elasticity in intact nuclei within living cancer cells. Our findings demonstrate that nuclear elasticity decreases when cells cultured without serum are exposed to serum or TGF-β. Notably, we found no significant changes in the expression levels of lamins A/C, B1, and B2 under these conditions. However, we observe a significant increase in H4K20me3 levels. These findings strongly suggest that chromatin compaction states influence nuclear elasticity. Exposure to serum or TGF-β reduces chromatin compaction, resulting in nuclear softening.

NE-AFM successfully mapped the elasticity distribution on the nuclear surface in living cells. The resulting elasticity map reveals highly heterogeneous distributions of nuclear elasticity (Figures 1k and 3c,e). The nuclear lamina has a meshwork structure with a pore size of 1–1.5 μm in diameter,^38^ and nuclear pore complexes (NPCs) are localized within these pores in the meshes.^73^ Heterochromatin tethered to the nuclear lamina forms lamina-associated domains (LADs),^74^ while regions near NPCs are enriched in euchromatin.^40^ It is reasonable to speculate that these structural features contribute to the heterogeneous distributions of nuclear elasticity. Further experiments are required to elucidate the precise relationship between nuclear elasticity and these underlying structures. Our method offers enhanced precision for such investigations.

In this study, we employed a modified Hertz model for a paraboloidal indenter to estimate the Young’s modulus from the force–indentation curves. We acknowledge that any simple analytical model represents an approximation for the specific geometry of a fabricated nanoneedle probe. Our probes have a tip with a radius of curvature of ∼24 nm, which then tapers, gradually widening to a diameter of ∼160 nm over a length of approximately 300 nm, before maintaining a constant shaft diameter. Although a few models are suggested for needle-shaped probes,^75^ there is no perfect analytical model for our specific probe geometry. Therefore, we chose a model that is both practical and consistent with the established precedent in the field of direct intracellular nanomechanical measurements.^27,28^ More importantly, the central conclusions of our manuscript are based on the relative changes in nuclear elasticity between different experimental conditions (i.e., with and without serum, and with TGF-β treatment). Any systematic error introduced by the choice of an imperfect-but-consistent model would be applied uniformly across all data sets. This error is therefore effectively canceled out when performing these relative comparisons. Thus, while the absolute Young’s modulus values should be interpreted within the context of this approximation, the observed trends—the stiffening upon serum depletion and softening after TGF-β exposure—and the biological insights drawn from them remain robust and valid.

To date, no reports have examined the effect of serum and TGF-β on nuclear elasticity. In this study, we demonstrated that nuclear elasticity increases under serum-depleted conditions compared to serum-containing conditions in both parental (PC9) and brain-metastatic (PC9-BrM) PC9 cells. We also observed that H4K20me3 levels increased in these serum-depleted cells. Previous studies reported that serum depletion increases H4K20me3 levels through G0/G1 phase arrest.^52,76^ The research group of Bierhoff et al. also reported that serum depletion causes an increase in H4K20me3 through the upregulation of long noncoding RNAs, which can recruit a histone methyltransferase (HMT) SUV4–20H2.^77^ These results support our model in which serum depletion increases H4K20me3 levels, leading to increased heterochromatin and, consequently, nuclear elasticity. We also investigated the impact of TGF-β on nuclear elasticity under serum-free conditions and found that the TGF-β treatment reduces nuclear elasticity in PC9 cells compared to control cells. Guerrero-Martinez’s group has reported that TGF-β upregulates microRNAs, miR-29a and miR-29c, through the activation of Smad. These microRNAs inhibit the translation of the HMTs Suv4–20h1 and Suv4–20h2, and consequently, H4K20me3 levels are reduced.^78^ These results strongly suggest that TGF-β promotes global chromatin relaxation, leading to a decrease in nuclear elasticity. Other factors, such as changes in the actin cytoskeleton induced by TGF-β treatment, could also affect nuclear elasticity. However, in our specific case, we found that neither the expression level of β-actin nor the amount of actin filaments in the area above the nucleus was significantly altered by TGF-β treatment (Supporting Information, Figure S2). Therefore, we conclude that the influence of the actin cytoskeleton on nuclear elasticity is likely minimal in our system.

When comparing nuclear elasticity between parental and brain-metastatic cells, we found that brain-metastatic cells (PC9-BrM) exhibit similar nuclear elasticity to parental PC9 cells. This result may seem unexpected, as EMT induction has been shown to decrease nuclear elasticity, and traversing the BBB is mediated by EMT.^79^ One possible explanation is that PC9-BrM cells may have undergone EMT temporarily to traverse the BBB but gradually lost their metastatic properties during the subsequent development of brain metastases, cell collection, and repeated passaging in plastic culture dishes.^80^ By the time nuclear elasticity was measured, PC9-BrM cells may have already lost their metastatic characteristics. On the other hand, cancer cells that underwent TGF-β-induced EMT directly are more likely to reflect EMT-associated properties.

The cell membrane elasticity of PC9-BrM cells exhibited opposite trends depending on the presence or absence of serum (Figure 2h); the cell membrane elasticity of PC9-BrM cells was significantly lower than that of PC9 in the presence of serum, whereas it was higher in its absence. This observation may help explain conflicting results on cancer cell elasticity measurements. For instance, one study reported that the elasticity of cervical cancer cells was higher than normal cells when both were cultured in keratinocyte serum-free medium (KSFM),^64^ while another found that cervical cancer cells cultured in a medium containing 10% fetal bovine serum were softer than normal cells cultured in KSFM.^81^ These findings suggest that differences in culture medium, particularly the presence or absence of serum, can significantly influence cell elasticity measurements. Therefore, we strongly recommend conducting all cell and nuclear elasticity measurements under controlled serum conditions.

The transition in the nuclear elasticity has been investigated as a potential biomarker for malignant cancer.^7,8^ Accurate measurement of nuclear elasticity could significantly enhance clinical assessment and diagnosis. The method developed in this study could become a fundamental technique in cancer diagnostics and has broader applications for measuring the mechanical properties of other intracellular structures. While the mechanical properties of structures within living cells remain largely unexplored, our technique can be applied to assess the elasticity and adhesion properties of other organelle structures, such as mitochondrial membranes or focal adhesions, in both healthy and diseased cells.^82,83^ Understanding these mechanical properties will provide deeper insights into nanoscale biology and the mechanisms underlying diseases associated with cellular dysfunction.^84^

## Methods

### Cell Sample Preparation

HeLa cells were obtained from the Japanese Collection of Research Bioresources (JCRB) cell bank (JCRB9004). PC9-Luc-EGFP and PC9-Luc-EGFP-BrM4 (brain-metastatic cells, referred to as PC9 and PC9-BrM, respectively, throughout the article) were established in a previous study.^57^ All cell lines were maintained in Dulbecco’s Modified Eagle’s Medium (DMEM, Gibco) supplemented with 10% fetal bovine serum (Biosera) and 1% penicillin/streptomycin (Fujifilm Wako).

### NE-AFM

PC9 and PC9-BrM cells were cultured on 35 mm plastic dishes in the DMEM (Fujifilm Wako) containing 10% serum, no serum, or no serum and 5 ng/mL TGF-β1 (Peprotech) for 2 days. Before observation, the culture medium was replaced with Leibovitz’s L-15 medium (Thermo Fisher Scientific) supplemented with 1% penicillin/streptomycin and containing either 10% serum, no serum, or no serum with 5 ng/mL TGF-β1. All experiments were conducted within a few hours after the medium change. The nuclear and cell membrane elasticities were measured using NE-AFM methods.^29–31^ Nanoneedle probes were fabricated using electron beam deposition with a focused ion beam system (Helios G4 CX Dual Beam, Thermo Fisher Scientific) on tip-truncated cantilevers (Olympus, BLAC40TS-C2, spring constant approximately 0.1 N/m). We used a JPK NanoWizard 4 BioAFM (Bruker) equipped with an inverted fluorescence microscope (Eclipse Ti2, Nikon). The temperature was maintained at 37 °C using a dish heater (Bruker). Measurements were conducted in QI mode with the following parameters: 16 × 16 pixels, 5.5–6.5 μm Z-length, 1.5 nN set point for whole-cell measurements or 0.6–0.8 nN for nuclear elasticity measurements, and 10 μm/s tip speed. For each sample, approximately 10 cells were measured. From the 256 force curves acquired per cell, 20 representative curves were selected for *E*_Y_ estimation. *E*_Y_ was calculated using custom software (Supporting Information, Figure S3). After applying a baseline correction to each force curve by subtracting the long-range, noncontact force, a fixed 0.1 nN range of the indentation segment was fit using the following equation (modified Hertz model for paraboloidal indenter).^14,48,49^ In this equation, F, Rc, ν, and δ indicate force, the probe’s radius, Poisson ratio, and indentation depth, respectively.^31^

$$F=\frac{4\sqrt{R_{c}}}{3}\frac{E_{Y}}{1-\nu^{2}}\delta^{3/2}$$

### Immunoblotting

Cultured cells on 10 cm dishes were washed three times with ice-cold PBS and lysed in 150 μL of RIPA or Laemmli buffer supplemented with protease inhibitor and phosphatase inhibitor cocktail (cOmplete and PhosSTOP, Roche). After lysis, samples were sonicated for 2 min, freeze–thawed once, and heated at 95 °C for 5 min. Protein concentration was measured using a BCA Protein Assay Kit (BioDynamics Laboratory), and samples were adjusted to equal concentrations. Samples were mixed with 0.005% bromophenol blue (Nacalai) and 125 mM DTT (Fujifilm Wako), then denatured at 95 °C for 5 min. Proteins (3–20 μg) were separated on 5–20% polyacrylamide gels (SuperSep Ace, Fujifilm Wako) with a marker (Precision Plus Protein, Bio-Rad) and transferred onto polyvinylidene fluoride (PVDF) or nitrocellulose membrane (Bio-Rad) using a Trans-Blot Turbo semidry transfer system (Bio-Rad). Membranes were blocked with Intercept Blocking Buffer (TBS; LI-COR) for 1 h at room temperature, incubated overnight at 4 °C with primary antibodies, and then incubated with secondary antibodies for 1 h at room temperature. Antibodies used for immunoblotting are listed in Supporting Table S2. Fluorescence signals were detected using an Odyssey CLx imaging system (LI-COR). Total proteins were visualized with Ponceau S Staining Solution (Supporting Information, Figures S4, Beacle), and images were analyzed using Image Studio Lite (LI-COR).

### Measurement of Cell Area and Nucleus Volume

Cells were cultured on a glass-bottom dish (ibidi). For measuring nuclear volume and nuclear deformation, the nuclei were stained with 1 μM SiR-DNA (Cytoskeleton). Fluorescence images were acquired in three dimensions using a confocal microscope (expert line, Abberior Instruments) with a resolution of 200 × 200 × 200 nm per pixel, using a 640 nm excitation and a 10 μs exposure time. Image analysis was performed using a custom MATLAB script (R2024a, Simulink). For measuring cell adhesion area, the cytosol was stained with 1 μg/mL Calcein-AM (Dojindo), and the cell membrane was stained with PlasMem Bright Green (1:1000 dilution, Dojindo). Scatter plots were generated using Prism 9 (GraphPad Software).

### Statistical Analyses

Statistical comparisons were performed using EZR software (Saitama Medical Center, Jichi Medical University).^85^ Welch’s *t*-test was used for single comparisons, while the Games-Howell test was used for multiple comparisons. *P*-values are indicated as follows: n.s. (nonsignificant, *p* > 0.05); * (*p* < 0.05); ** (*p* < 0.01); *** (*p* < 0.001); and **** (*p* < 0.0001).

All other analyses, including histogram generation, Gaussian fitting, and the calculation of standard deviations and coefficients of determination, were performed using Prism 9.

### F-Actin Imaging

Cells were cultured on 35 mm glass-based dishes (Matsunami), fixed with 4% paraformaldehyde (Electron Microscopy Sciences) for 10 min, and then permeabilized with 0.1% Triton X-100 (Nacalai Tesque) in PBS for 15 min. Fixed cells were incubated for 1 h at room temperature with 1 μM Spirochrome SiR-Actin probe (Cytoskeleton, Inc.) in PBS. Following incubation, cells were counterstained with Hoechst 33,342 (Nacalai Tesque) to visualize DNA. The F-actin cytoskeleton was imaged using a Dragonfly spinning disk confocal microscope system (CR-DFLY-301; Andor, an Oxford Instruments Company) equipped with Plan-Apochromat λD 100× (NA 1.45) oil-immersion objective lens. Images were acquired using an iXon Life 888 EMCCD (25% 405 nm laser transmission; 2% 637 nm laser transmission; 1024 × 1024 pixels; 40 μm pinhole; 2-frame averaging) operated by Fusion software (v. 2.4.0.22). Max intensity projection images, which were generated from z-stacks acquired at 0.13 μm intervals, were analyzed using Imaris 9.3.1 (Bitplane, an Oxford Instruments Company).

## Supplementary Material



## Notes

Figure 1: Effect of the bottom effect correction on calculated Young’s modulus maps and distributions. Figure 2: β-actin and F-actin expression Figure 3: procedure for fitting force–distance curves to determine nuclear elasticity. Figure 4: total protein bands from a Western blot. Supporting Table 1: Young’s modulus of the nuclear surface and cell membrane under serum-present (Serum+), serum-absent (Serum−), and TGF-β-treated conditions. Table 2: Antibody list used for immunoblotting (PDF)
