# Supplementary material for: Probing Nanomechanics by Direct Indentation Using Nanoendoscopy-AFM Reveals the Nuclear Elasticity Transition in Cancer Cells
Source: ACS Appl Nano Mater. 2025 Oct 12;8(42):20239–49. doi: 10.1021/acsanm.5c03044 (PMC12560078; doi:10.1021/acsanm.5c03044)
Supplement: Supplementary file 1 [file an5c03044_si_001.pdf]

Supporting Information for

Probing nanomechanics by direct indentation using Nanoendoscopy-AFM reveals the nuclear elasticity transition in cancer cells.

Takehiko Ichikawa[1], Yohei Kono[1], Makiko Kudo[1], Takeshi Shimi[1], Naoyuki Miyashita[2], Tomohiro Maesaka[2], Kojiro Ishibashi[3], Kundan Sivashanmugan[4], Takeshi Yoshida[1,5], Keisuke Miyazawa[1,2], Rikinari Hanayama[1,5], Eishu Hirata[1,3], Kazuki Miyata[1,2], Hiroshi Kimura[6,7], Takeshi Fukuma[1,2]**

[1] Nano Life Science Institute (WPI-NanoLSI), Kanazawa University, Kakuma-machi, Kanazawa, Ishikawa, 920-1192, Japan

[2] Division of Electrical Engineering and Computer Science, Kanazawa University, Kakuma-machi, Kanazawa, Ishikawa, 920-1192, Japan

[3] Division of Tumor Cell Biology and Bioimaging, Cancer Research Institute of Kanazawa University, Kakuma-machi, Kanazawa, Ishikawa, 920-1192, Japan

[4] Biochemistry and Molecular Biology, University of Maryland School of Medicine, 655 W. Baltimore Street, Baltimore, MD, 21201

[5] Department of Immunology, Kanazawa University Graduate School of Medical Sciences, Kakuma-machi, Kanazawa, Ishikawa, 920-1192, Japan

[6] Cell Biology Center, Institute of Integrated Research, Institute of Science Tokyo, 4259 Nagatsuta-cho, Midori-ku, Yokohama, 226-8501, Japan

[7] Graduate School of Bioscience and Biotechnology, Institute of Science Tokyo, 4259 Nagatsuta-cho, Midori-ku, Yokohama, 226-8501, Japan

*Corresponding author

E-mail address

Takehiko Ichikawa: tichikawa@staff.kanazawa-u.ac.jp

Takeshi Fukuma: fukuma@staff.kanazawa-u.ac.jp

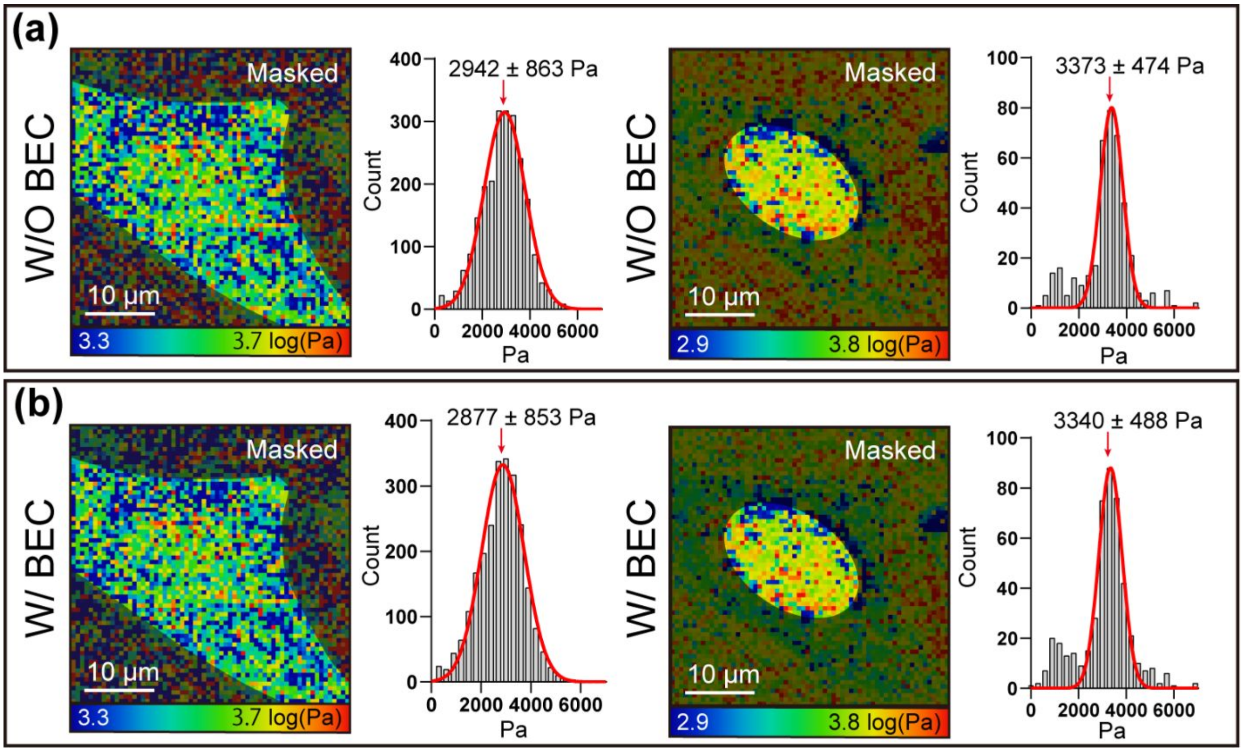

Figure S1. Effect of the bottom effect correction on calculated Young's modulus maps and distributions. (a) Elasticity data calculated without the bottom effect correction. Shown from left to right are: the Young's modulus (E_Y) map of the cell membrane, the corresponding histogram of elasticity values (peak value: 2942 ± 863 Pa), the E_Y map of the nucleus, and its corresponding histogram (peak value: 3373 ± 474 Pa). (b) The same data as in (a), but calculated with the bottom effect correction applied (peak values: 2877 ± 853 Pa in cell membrane, 3340 ± 488 Pa in nucleus). Note the minimal change in the maps and the overall distributions.

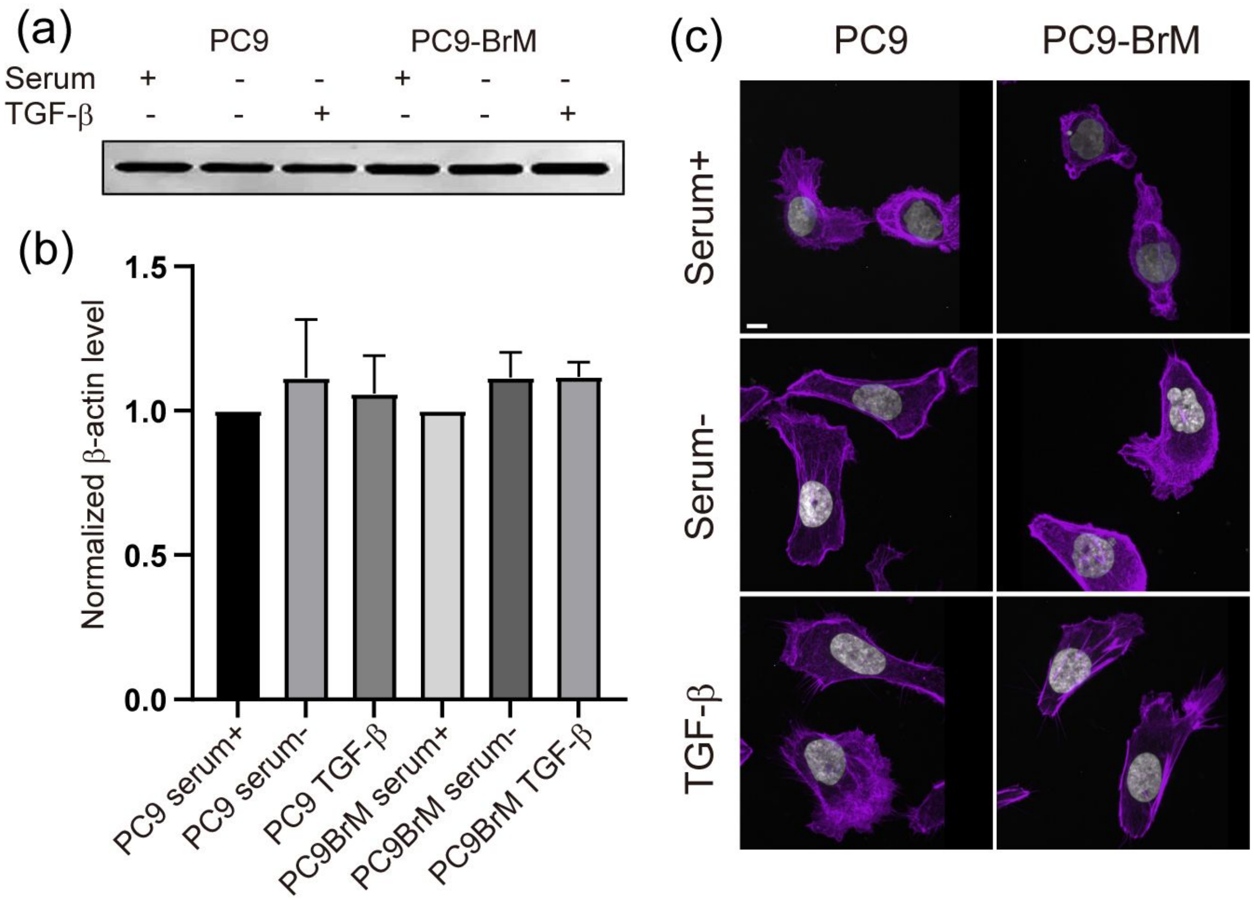

Figure S2. β-actin and F-actin expression (a) Representative immunoblot showing β-actin expression in PC9 and PC9-BrM cells under the indicated treatment conditions (serum-containing, serum-depleted, and TGF-β-treated). (b) Quantification of β-actin expression. Each bar was normalized to the expression level under serum-containing (serum+) conditions for PC9 and PC9-BrM cells, respectively.(c) Representative fluorescence images of F-actin (magenta) and nuclei (white). Scale bar: 7 μm.

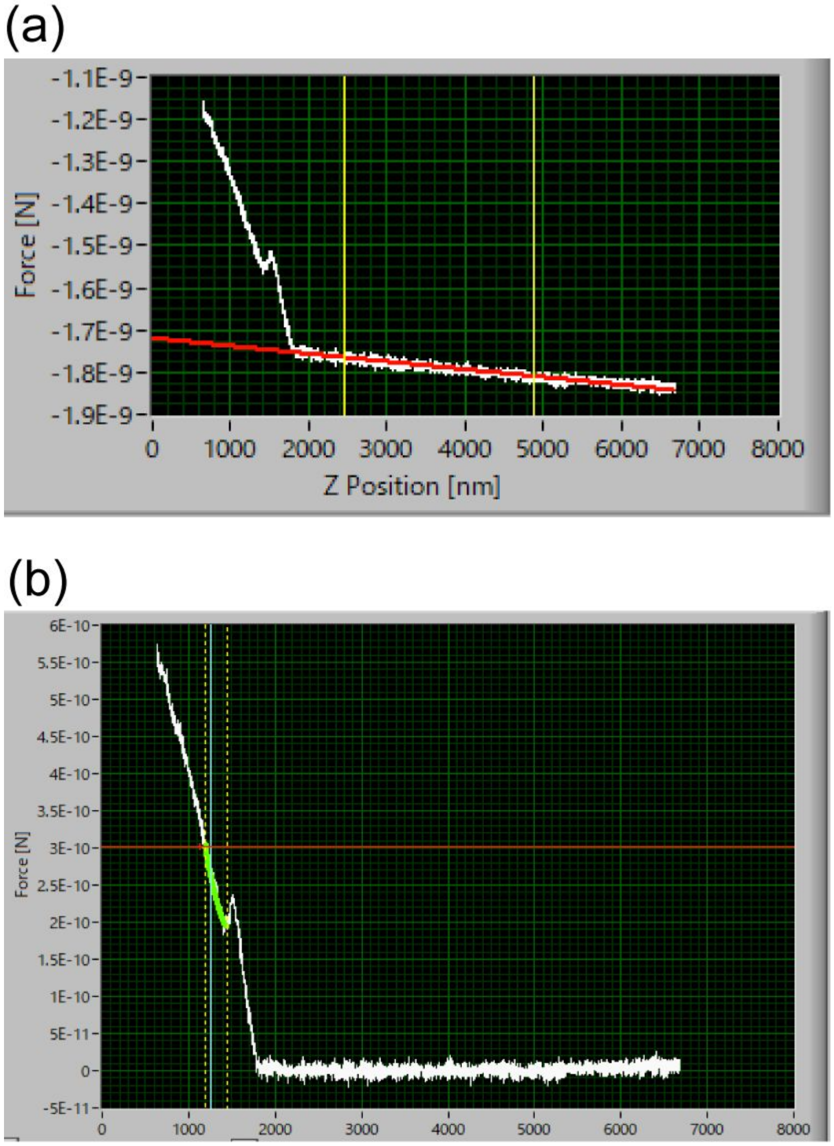

Figure S3. Procedure for fitting force-distance curves to determine nuclear elasticity. This analysis was performed using custom-written software[1]. (a) Baseline correction of the force curve. (b) Force curve fitting of the nuclear indentation segment. The optimal fitting region was automatically identified by sliding a 0.1 nN force window along the Z-axis. The Young's modulus was then calculated from this region using a modified Hertz model for a paraboloidal indenter (diameter: 30 nm).

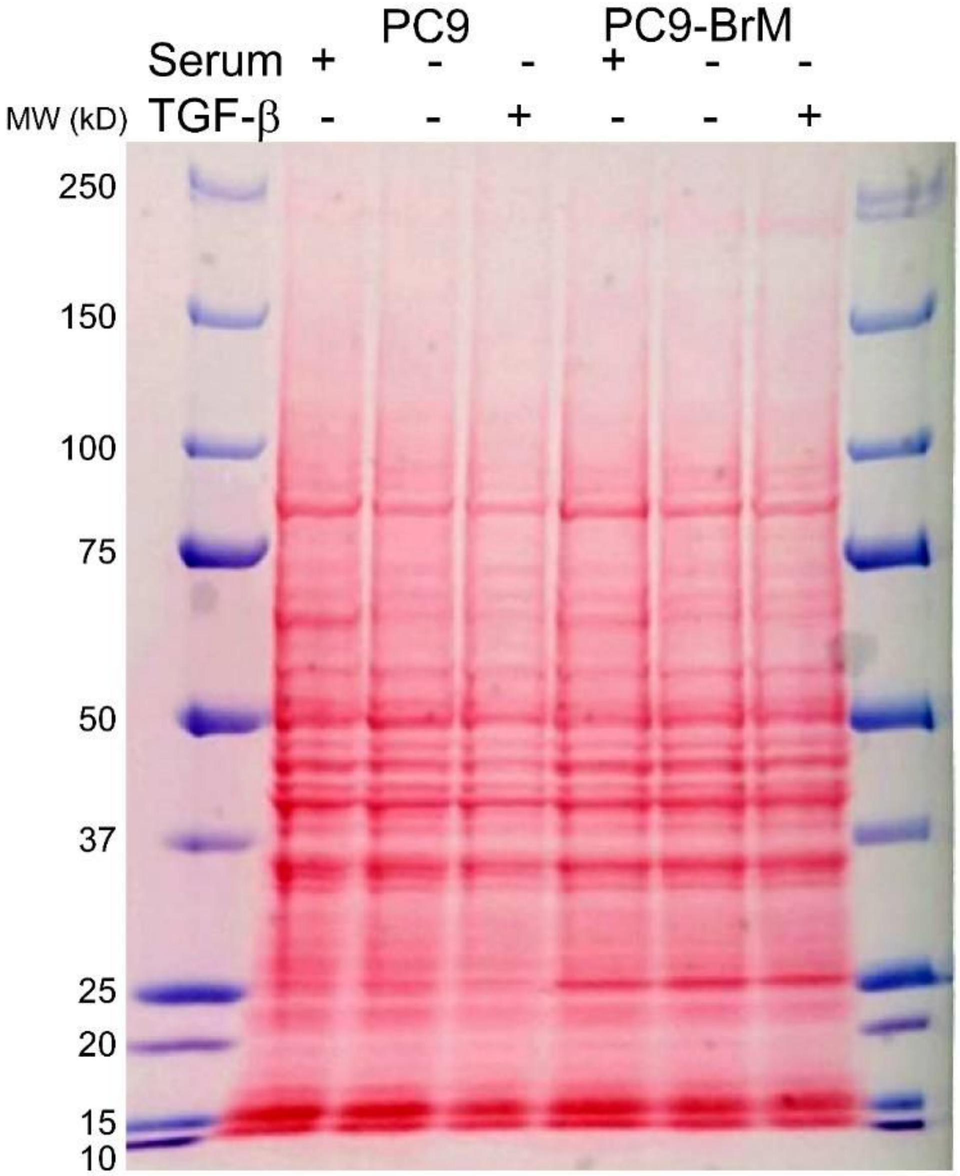

Figure S4. Total proteins stained with Ponceau S related to Figures 2f and 3f. MW: molecular weight.

Pa ± SEM	Serum+	Serum-	TGF-β
PC9 nucleus	2714 ± 126	4384 ± 284	3201 ± 198
PC9-BrM nucleus	2394 ± 249	4122 ± 269	
PC9 cell membrane	4722 ± 250	4166 ± 198	4097 ± 302
PC9-BrM cell membrane	2929 ± 241	6371 ± 477	

Supporting Table 1. Young's modulus of the nuclear surface and cell membrane under serum-present (Serum+), serum-absent (Serum-), and TGF-β-treated conditions.

Primary antibody				
Target	Host	Clone/Name (Source)	RRID	Dilution rate
Vimentin	Rabbit	D21H3 (Cell Signaling)	AB_10695459	1:500
N-cadherin	Mouse	13A9 (Santa Cruz)	AB_781744	1:200
Lamin A/C	Mouse	3A6-4C11 (eBioscience)	AB_2802197	1:20,000
Lamin B1	Rabbit	12987-1-AP (Proteintech)	AB_2136290	1:6,000
Lamin B2	Mouse	8F6-E8-F12 (Biolegend)	AB_2832882	1:1,000
H4K20me3	Mouse	CMA423/27F10[2]		1:6,000
Pan-H4	Mouse	CMA400/9C5[2]		1:5,000
Secondary antibody				
Name (Source)			RRID	Dilution rate
IRDye 800CW Donkey anti-Mouse IgG (LI-COR)			AB_621847	1:20,000
IRDye 680RD Donkey anti-Rabbit IgG (LI-COR)			AB_10954442	1:20,000

Supporting Table 2. Antibody list used for immunoblotting.

References

(1) Ichikawa, T.; Alam, M. S.; Penedo, M.; Matsumoto, K.; Fujita, S.; Miyazawa, K.; Furusho, H.; Miyata, K.; Nakamura, C.; Fukuma, T. Protocol for live imaging of intracellular nanoscale structures using atomic force microscopy with nanoneedle probes. *STAR Protoc* **2023**, *4* (3), 102468. DOI: 10.1016/j.xpro.2023.102468 From NLM Medline.

(2) Kontomaris, S.; Malamou, A. Hertz model or Oliver & Pharr analysis? Tutorial regarding AFM nanoindentation experiments on biological samples. *Materials Research Express* **2020**, *7* (3), 033001.
